# Editorial: Auditory Efferent System: New Insights from Cortex to Cochlea

**DOI:** 10.3389/fnsys.2016.00050

**Published:** 2016-06-07

**Authors:** Paul H. Delano, Ana B. Elgoyhen

**Affiliations:** ^1^Programa de Fisiología y Biofísica, Instituto de Ciencias Biomédicas (ICBM), Facultad de Medicina, Universidad de ChileSantiago, Chile; ^2^Departamento de Otorrinolaringología, Hospital Clínico de la Universidad de ChileSantiago, Chile; ^3^Facultad de Medicina, Instituto de Investigaciones en Ingeniería Genética y Biología Molecular, Dr. Héctor N. Torres (INGEBI)Consejo Nacional de Investigaciones Científicas y Técnicas (CONICET) Buenos Aires, Argentina; ^4^Tercera Cátedra de Farmacología, Universidad de Buenos AiresBuenos Aires, Argentina

**Keywords:** auditory efferent, olivocochlear, top-down, corticofugal, outer hair cells

The outer hair cells (OHCs) of the mammalian cochlea are unique. In average, there are about 15,000 mechanical receptor cells in each human cochlea, and OHCs constitute three quarters of them. They possess the cellular machinery to achieve two fundamental physiological mechanisms of the nervous system: mechanotransduction and electromotility. Through the process known as electromotility, OHCs convert membrane voltage changes into mechanical force. Electromotility depends on a unique motor protein called prestin and results in important auditory functions such as cochlear amplification and fine tuning of the cochlear basilar membrane. The OHCs receive efferent projections from the central nervous system through medial olivocochlear neurons (MOC), located in the brainstem. MOC neurons release acetylcholine which activates unique nicotinic receptors constituted by α9/α10 subunits. In addition to the olivocochlear system, there are massive descending projections from the auditory cortex to the thalamus, inferior colliculus, cochlear nucleus, and superior olivary complex. All these descending pathways could modulate cochlear responses through olivocochlear neurons. Taken together, the corticofugal projections and the olivocochlear system constitute a complex efferent neural network from cortex to the cochlear receptor that regulates synapses between MOC and OHC.

In this e-book, entitled “Auditory efferent system: new insights from cortex to cochlea,” we aimed to give an overview of the advances concerning the descending projections from the auditory cortex to subcortical nuclei and the olivocochlear system. In addition, different theoretical proposals of efferent functions are presented.

Guinan gives an expert comprehensive review of the different methods used to assess the olivocochlear function in human and animal experiments. One key aspect discussed in this perspective article is the need to separate crossed and uncrossed MOC effects on otoacoustic emissions. Moreover, more rigorous statistical analyses, like bootstrap methods are proposed.

Aguilar et al. used contralateral acoustic stimulation with broad-band noise in humans and measured behavioral consequences on pure tone thresholds. They found that MOC activation was variable among subjects, as it increased the absolute detection thresholds in some individuals but not in others. Their results suggest that the intensity range of the mechanical suppression produced by MOC activation in humans is different for 500 and 4000 Hz tones.

Otoacoustic emissions have been widely used as a non-invasive tool to evaluate the olivocochlear reflex in animals as well as in humans. Zhao et al. used broad-band noise to assess MOC function with stimulus frequency otoacoustic emissions. Similar to distortion product otoacoustic emissions, they found a shift toward higher frequencies with contralateral acoustic stimulation. These results suggest that the effects of contralateral broad-band noise stimulation on otoacoustic emissions and hearing thresholds are driven by a common set of mechanisms.

Aedo et al. show that suppression of the compound action potential of the auditory nerve with contralateral acoustic stimuli are larger in awake chinchillas compared to anesthetized animals. Moreover, the frequency selectivity of contralateral suppression for tones above 4 kHz is shifted around ¼ octave from the ipsilateral tone. These results show that the strongest olivocochlear effects are produced by contralateral tones at frequencies equal or close to those of ipsilateral tones.

Katz and Elgoyhen present an enlightening review on medial olivocochlear synapses of cochlear hair cells. They discuss how synaptic plasticity mechanisms regulate the efficacy of these synapses at the pre and postsynaptic levels.

Smith and Keil propose an interesting debate about the evolutionary role of the medial olivocochlear system. Loud noises in natural environments are infrequent and consequently it seems improbable that the olivocochlear system evolved to protect against high intensity sounds. They argue that in a biological context, probably the main function of the efferent system is to increase the signal-to-noise ratio during detection of target signals.

On the other hand, Fuente presents a systematic mini-review of the role of MOCs for protection from acoustic trauma in animal models and in human subjects. Animal experiments have clearly shown that olivocochlear fibers can protect from acoustic injuries, however human data is still contradictory and more research is needed.

Terreros and Delano review recent exciting findings showing that auditory cortex activity modulates the most initial auditory processing, including the cochlea, auditory nerve, and cochlear nucleus. In addition, a working model for the different loops of the corticofugal auditory pathways is proposed.

Mellott et al. used retrograde and anterograde tracers to demonstrate that corticofugal projections from the auditory cortex contact excitatory and inhibitory cells in the nucleus of the brachium of the inferior colliculus. Importantly, these findings evidence complex interactions in the efferent loops at the midbrain level.

Malmierca et al. review one key aspect of brain functioning such as the role of corticofugal projections in sensory adaptation. Notably, they evidence that stimulus sensory adaptation in the midbrain is modulated by the corticofugal pathways, and propose a model of predictive coding in the auditory system.

Kong et al. use electrical microstimulation of the auditory cortex to study corticofugal projections to the dorsal cochlear nucleus in mice. These authors describe that the corticofugal effects on the dorsal cochlear nucleus are frequency specific, similar to the corticofugal modulation of ventral cochlear nucleus.

Finally, Lamas et al. design an original approach to investigate the influences of auditory cortex on prestin expression in OHCs. They report changes in prestin oligomerization after surgical removal of the auditory cortex, suggesting that the corticofugal pathways could modulate OHC electromotility.

Some of the unknown fields that should be addressed by future research in the auditory efferent system include physiology of the cortico-olivocochlear neural dynamics, establishment of a reliable and non-invasive clinical measure of the olivocochlear reflex strength in humans and pharmacological development of selective drugs for human use.

## Author contributions

All authors listed, have made substantial, direct and intellectual contribution to the work, and approved it for publication.

### Conflict of interest statement

The authors declare that the research was conducted in the absence of any commercial or financial relationships that could be construed as a potential conflict of interest.

